# Newspaper Charlatanism

**Published:** 1875-08

**Authors:** 


Newspaper Charlatanism.—Of all the manifestations and implements of the medical charlatan of the present day, there are none so dangerous and disgraceful as those connected with the daily newspaper press. Indeed, in every city, town and village there are a certain number of physicians who are thoroughly well known to use this venal agent as the basis of notoriety and emolument. They have planted their seed carefully and secretly; they have enriched the ground by constant expenditures for costly fertilizers; and they have largely reaped the harvest which springs from the culture of such seed. Their barns are full; their fields are teeming, and like the rich man of Palestine, they fancy that they will continue to buy, and get much gain. But as their rich prototype was cut down in the midst of his plentitude, so will a similar fate be theirs; for, sooner or later, they too will be summoned before a dread tribunal—that remorseless tribunal of physicians, professional sentiment, to meet a just retribution for their long years of personal manœuvering and professional degradation.

Who does not know each newspaper charlatan in his neighborhood? How plausible he is; how he abuses the tricky use of the press by others; how devoted he is to medical societies; how professionally upright and personally frank and ingenuous. He knows every editor and sub-editor; he calls them all by name; indeed, by their Christian name; more than this, each Christian name is pleasingly abbreviated, and "Tom.," "Bal,," "Hal." etc. are truly happy or amused over his cordial warmth, familiarity and frankness. The managing or chief editor finds in his sanctum a brace of pheasants, or a choice string of partridges, or snipe, etc., "with the compliments of Y." or B. Choice "Bourbon" or a box of the best cigars, is deposited, with a similar pleasing card, upon the desk of the sub-editor, "the local." Pleasant rides, *petit soupers*, charming games of cards, "with a few intimates only," are furnished with the rarest judgment. Indeed, the editors are handled with as much skill as Isaac Walton ever displayed with the reel and line, and like the simplest fish, they are hooked and landed with the most charming profit and unfailing dexterity. And now for the reward. What charming little paragraphs and delicate eulogies; how naturally appear professional essays and extracts from periodicals and choice editorial notices culled from distant newspapers, whose editors have been equally simple and equally well managed; what splendid notices, elsewhere obtained and conspicuously marked, come in a flood into the editor's sanctum; and what princely editions of every issue in which these notices re-appear are purchased and judiciously distributed; how many inflnential medical friends have their nice little medical essays published and profitably disseminated; and what a glorious time all parties have over the matter; and how the editor's folly is well known to all; and how the miserable charlatans, behind the screen as they suppose, are invariably understood and weighed in private colloquy, and ridiculed and despised.

The reader may say: well, why publish all these facts; they are true and well known to physicians, but what can be done; what good can be accomplished by publishing them? Those who say this have not watched the results of such publications in medical journals. The culprits are not confessed and open charlatans; they claim society and professional position; they dread exposure and being held up naked and in derision before the great body of that profession whom they degrade and disgrace, but whom they fear with an abject terror. Secrecy is success and retained prosperity; exposure is failure and ruin. These men may affect to laugh at the medical press, but they know its power, and fear it; they know it can unmask and destroy them; and laugh as they often do, they watch it closely, and feel that it can punish if it cannot reform them. When the Queen of Navarre was asked not to heed the lays of the troubadour, she wisely said: "You are wrong; let me see the oracle that can tell nations what I am;" and however silly and rollicking bravado may affect to disregard the press, the silent monitor within repeats in effect, with increasing and successful force, these truthful words of the wise Queen.—*American Medical Weekly.*

